# Communication during out-of-hours primary care contacts for people with a terminal illness: a scoping review

**DOI:** 10.1136/bmjopen-2025-105738

**Published:** 2026-02-27

**Authors:** Rebecca Anderson-Kittow, Roisin Dillon, Rebecca K Barnes

**Affiliations:** 1 Nuffield Department of Primary Care Health Sciences, University of Oxford, Oxford, UK

**Keywords:** PALLIATIVE CARE, Primary Health Care, Decision Making

## Abstract

**Abstract:**

**Objective:**

To summarise what is known about communication during out-of-hours primary care contacts for people with a terminal illness.

**Design:**

A scoping review following the Joanna Briggs Institute guidance for scoping reviews and conducted in accordance with Arksey & O’Malley’s methodological framework for scoping reviews.

**Data sources:**

Searches of MEDLINE, PsycINFO, CINAHL and EMBASE were conducted from inception to 23 July 2024, alongside grey literature searches and hand searching reference lists of relevant reviews.

**Eligibility criteria:**

Sources were eligible if they provided evidence about communication between people with a terminal condition, their families and/or healthcare professionals during contacts with out-of-hours primary care services.

**Data extraction and synthesis:**

Data were extracted by two independent researchers following Joanna Briggs Institute guidelines for scoping reviews. Findings were thematically synthesised to create a narrative account of the evidence.

**Results:**

Of the 1745 records identified, 18 studies were included in the review. Most used qualitative interviews and/or focus groups. Barriers to good communication included a lack of continuity of care, problems relating to remote consultations, the delegitimising of help seeking, and the challenges of conducting specialist palliative care consultations within a generalist out-of-hours system. Facilitators to good communication included the availability of information about patients and families out of hours, an empathetic and confident approach from out-of-hours professionals, and support from colleagues.

**Conclusions:**

The scoping review showed that there is limited research focusing specifically on end-of-life communication in out-of-hours primary care settings. Further research is needed, particularly using observations or recordings of real interactions. There are several challenges to communication in this setting, but providing clinicians with access to palliative care summaries, alongside training and support in this specialised communication, can facilitate good end-of-life communication with patients and their families.

**STRENGTHS AND LIMITATIONS OF THIS STUDY**

- A protocol for the scoping review was registered on Open Science Framework in advance of searches being conducted.
- The scoping review approach was systematic and enabled findings from studies with different research designs, aims and research questions to be synthesised.
- Patient and public involvement representatives were not consulted during protocol development as the group had not been formed at that point, but were consulted during the interpretation and presentation of the findings.
- No quality appraisal was conducted as this would have been inconsistent with the aim of our scoping review.
- Despite comprehensive database searching, relevant studies may have been missed, particularly non-English language sources. Only 40% of titles, abstracts and full texts were screened by a second reviewer.

## Introduction

 The proportion of people dying at home is increasing and this trend is anticipated to continue.^1^ A key part of care in the community is out-of-hours (OOH) primary care; around one-third of patients had contacts with OOH primary care services within 30 days of their death.^2^ In the UK, for most people, access to such services usually involves contacting a national urgent medical help service. In England, this involves contacting NHS 111 or direct professional-to-professional lines (eg, a district nurse calling the general practitioner (GP) OOH service for advice about a patient). If necessary, this is followed by a callback with a clinician who will assess the situation and either provide advice only, a face-to-face appointment or onward referral. Primary care is often the first point of contact for patients OOH and sits at the interface between specialist palliative care, secondary care and emergency services. Good communication in OOH primary care is therefore vital to patients receiving appropriate care. Getting palliative and end-of-life care right is important to ensure patients’ pain, fatigue, nausea, agitation and other palliative symptoms are treated swiftly, unnecessary hospitalisations are avoided, and timely conversations are had to prepare patients and families for the patient’s death. A systematic review found that poor communication contributed to end-of-life hospital admissions OOH.^3^

Communication is reported as a key factor in patients’ and families’ satisfaction with both end-of-life and OOH care.^4^,^6^ OOH care featured in the top ten research priorities identified by the Palliative and end of life care Priority Setting Partnership in both 2015 and 2025, and communication was a key theme throughout survey responses.^7^,^9^ OOH, the community context poses particular communication challenges for healthcare professionals, patients and families. Since 2004, GPs have not been contractually required to provide OOH services to patients themselves. These services are now provided by large private companies, social enterprises and NHS Trusts. These organisations are staffed by non-clinical call handlers and healthcare professionals who are unlikely to have met the patient before.

Communication with other professionals is also important in this context. A high proportion of terminal care calls to OOH services come from district nurses, nursing home staff and other healthcare professionals. Good working relationships and communication between professionals can help to avoid hospitalisations, for example, for nursing home residents.^10 11^ OOH clinical staff themselves may also seek advice from specialist palliative care services (eg, hospice advice lines) and so awareness of these services and clear communication during these calls are important.^12^

To improve contacts with people with a terminal illness and their families OOH, we need an understanding of the barriers and facilitators to good communication. The aim of this scoping review is to synthesise the literature regarding communication during OOH contacts with primary care services for people with a terminal condition and identify any gaps. It does so by addressing the following questions:

What is known about communication during contacts between people with a terminal illness, their family members and/or healthcare professionals in OOH primary care settings?

1. Which participant groups have been included (ie, patients, families, healthcare professionals)?
2. Which methods have been used to investigate communication during these contacts?
3. What is known about the barriers and facilitators to good communication?

## Method

A scoping review method was selected as its strengths lie in synthesising findings from different research designs and identifying gaps in understanding and areas where little research has been done.^13^

The Joanna Briggs Institute (JBI) guidance for scoping reviews^14^ was followed and a protocol based on the JBI template for scoping review protocols^15^ was registered on the Open Science Framework (https://osf.io/dncfa). The review was conducted in accordance with Arksey & O’Malley’s^13^ methodological framework for scoping reviews. The Preferred Reporting Items for Systematic Reviews and Meta-Analyses (PRISMA) Extension for Scoping Reviews (PRISMA-ScR) checklist and guidelines for reporting scoping reviews were followed.^16^

### Search strategy

**Figure 1:**
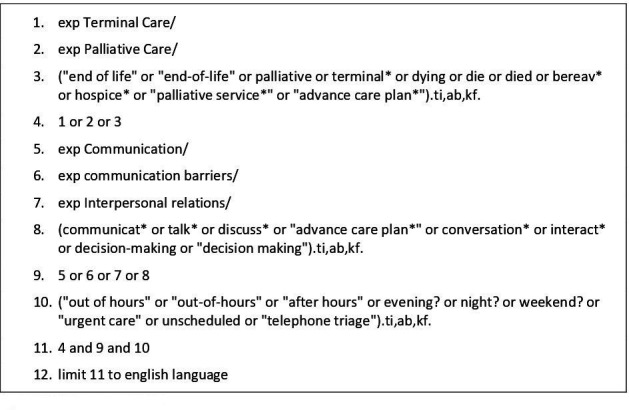
MEDLINE search terms.

Terms in titles and abstracts of relevant articles and search terms from relevant reviews were used to identify keywords and index terms, and develop a full search strategy with support from an information specialist. The following databases were searched on 23 July 2024, with search terms adapted for the different databases: MEDLINE, PsycINFO, CINAHL, EMBASE. The searches covered three domains: communication, end-of-life and OOH. There were no search restrictions based on date of publication. The full search strategy for MEDLINE is shown in figure 1 and the full search strategies for all databases are provided in online supplemental appendix A.

Reference lists of all included papers and five relevant reviews^17^,^21^ were handsearched. Grey literature was searched through ProQuest and hand searching relevant charity and clinical association websites (eg, Marie Curie).

### Eligibility criteria, study selection and data extraction

Participants: Patients receiving palliative care or approaching the end of life, family members or others accompanying these patients, and/or professionals working with these patients. End of life is not always recognised or predictable,^22^ and prognosis is often unreported in publications. Therefore, eligibility was not limited by prognosis; any papers in which the authors define patients as receiving palliative care or approaching the end of life were included. If a range of professionals were included and it was not possible to separate findings for primary care clinicians, at least 50% must be primary care clinicians. Other clinicians (eg, paramedics) were included if they were interacting with primary care clinicians.

Concept: Studies including some detail about experiences and/or observations of communication (verbal, non-verbal or written) between patients, companions and/or professionals during end-of-life OOH primary care. Studies focusing on communication outside of the OOH consultation (eg, written handover forms) were only included if their impact on communication during the consultation was described.

Context: OOH primary care services (telephone advice services, appointments at primary care centres and home visits).

RA-K ran the searches, removed duplicates and screened titles, abstracts and full texts using the review management platform, Rayyan. RD screened 40% of titles, abstracts and full texts. Disagreements were resolved through discussion.

Following the JBI guidance, only concepts and findings relevant to the research questions (ie, experiences of communication in OOH primary care at the end of life, the barriers and facilitators to good communication, and the approaches taken to research this topic) were extracted.^14^ RA-K and RD completed data extraction for all included papers.

### Data synthesis

An overview of study characteristics gave an understanding of the current state of the literature. Findings, including results and discussion sections of papers, were entered into NVivo V.15. Thematic synthesis, following Thomas and Harden’s^23^ approach, was used to synthesise qualitative findings. This involved line-by-line coding of relevant findings, followed by grouping codes into descriptive themes. These inductive, descriptive themes were then considered with the review questions in mind (ie, identifying barriers and facilitators of good communication) and analytical themes were developed to create a narrative account of the findings. Scoping reviews do not require a quality appraisal of included studies and so all papers were given equal weight during the synthesis.^13 14^

### Patient and public involvement

Preliminary findings were presented to a group of three people with experiences of OOH care for patients approaching end-of-life as family members. Initial themes were discussed in the context of their own experiences of OOH end-of-life care. This helped to validate the analysis and provided a guide for areas to look at in more detail. For instance, the theme ‘Out-of-hours professionals manner’ was developed as a theme in its own right after patient and public involvement (PPI) representatives emphasised that this was at the core of how positively or negatively they recalled their experiences of OOH communication. They also highlighted the value of including quotes from participants to make the findings clearer and more powerful.

## Results

**Figure 2:**
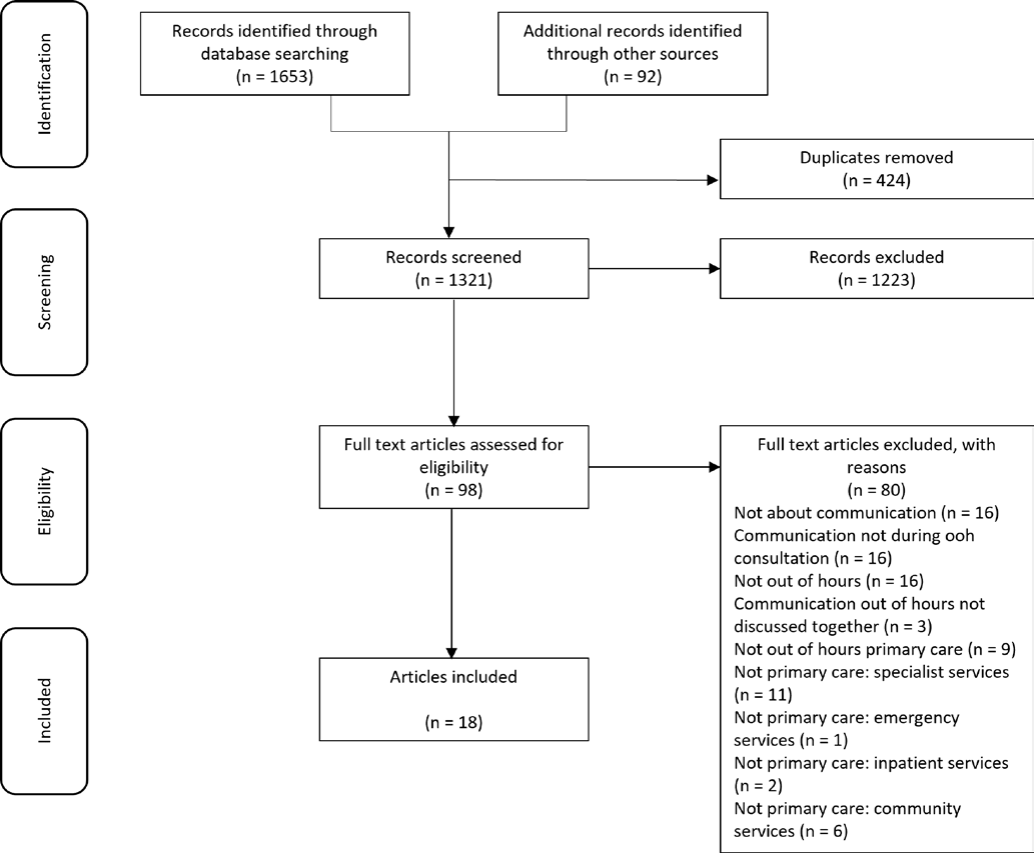
PRISMA diagram. PRISMA, Preferred Reporting Items for Systematic Reviews and Meta-Analyses.

The search returned 1653 records with a further 92 identified from other sources. 18 articles were included in the final review (see figure 2). A summary of included papers is presented in table 1 and a more detailed summary of findings is provided in online supplemental appendix B. Results are presented below, organised by the research questions outlined in the introduction.

**Table 1:** Summary of included papers

Author (date)	Country	Population	Focus of relevant evidence	Methods*
Adam *et al* (2015)^31^	Scotland	11 patients 4 caregivers	Communication between professionals and patients/families	Semistructured interviews
Asprey *et al* (2013)^32^	England	8 patients/carers 15 professionals	Communication between professionals and patients/families	Action research: Interviews and focus groups
Badger *et al* (2012)^34^	England	Survey: 79 nursing homes (49 completed both presurveys and postsurveys) Interviews/focus groups: 65 professionals	Communication between different professionals	Mixed methods: preprogramme and postprogramme surveys and case studies using interviews and focus groups
Fergus *et al* (2010)^38^	Scotland	6 patients 1 carer 29 professionals	Communication between different professionals Communication between professionals and patients/families	Rapid participatory appraisal: interviews and observations
Hall *et al* (2012)^39^	Scotland	2 patients 4 carers 16 professionals	Communication between different professionals Communication between professionals and patients/families	Semistructured interviews
Hanratty *et al* (2014)^40^	England	30 patients 118 bereaved caregivers 43 professionals	Communication between different professionals Communication between professionals and patients/families	Interviews (Routine data also used but did not contribute to findings on communication)
King *et al* (2004)^26^	England	15 bereaved carers	Communication between professionals and patients/families	Semistructured interviews
Leydon *et al* (2013)^24^	England	22 patients 4 carers (2 current, 2 bereaved)	Communication between professionals and patients/families	Prospective interviews
Lloyd-Williams and Rashid (2003)^33^	England	98 calls from professionals	Communication between different professionals	Audit
Mason *et al* (2022)^29^	Scotland	50 patients/carers (current and bereaved) 8 professionals	Communication between professionals and patients/families	Prospective interviews and focus groups
Richards *et al* (2011)^27^	England	13 patients 15 caregivers	Communication between professionals and patients/families	Semistructured interviews
Schweitzer *et al* (2011)^35^	Netherlands	20 professionals	Communication between different professionals Communication between professionals and patients/families	Focus groups
Seamark *et al* (2014)^25^	England	59 bereaved carers	Communication between professionals and patients/families	Semistructured interviews
Taubert and Nelson (2010)†^36^	Wales	9 professionals	Communication between different professionals Communication between professionals and patients/families	Semistructured interviews
Taubert and Nelson (2010)†^37^	Wales	9 professionals	Communication between different professionals Communication between professionals and patients/families	Semistructured interviews
Taubert *et al* (2011)†^28^	Wales	9 professionals	Communication between professionals and patients/families	Semistructured interviews
Thomas (2009)^41^	England	Stakeholder workshops: 20 professionals; 1 patient Surveys: 39 professionals	Communication between different professionals Communication between professionals and patients/families	Whole system participatory action research using stakeholder workshops and a survey
Worth *et al* (2006)^30^	Scotland	39 patients 67 carers 50 professionals	Communication between professionals and patients/families	In-depth interviews and focus groups

* Only methods used to collect relevant evidence included.

† Papers based on the same dataset.

### Which participant groups have been included?

A total of 124 patients, 287 family members/informal carers, a further 58 patients/carers (not reported separately in the papers) and 485 professionals (including GP, nurses, practice managers, care staff, palliative care specialists, commissioners and administrators) participated in the included studies. Nine papers included findings about communication between professionals and patients/families, four of which had patients/families only as participants,^24^,^27^ one had professionals only,^28^ and four included both patients/families and professionals.^29^,^32^ Two papers included findings on communication between different professionals, both of which had professionals only as participants.^33 34^ Seven papers covered both communication between professionals and patients/families and communication between different professionals, three of which included professionals only as participants^35^,^37^ and four included both patients/families and professionals.^38^,^41^

### Which methods have been used to investigate communication during these contacts?

17 out of the 18 included papers used qualitative or mixed methods. 15 papers used qualitative methods only (14 using interviews and/or focus groups^24^,^3235^ and one combining these with observations^38^); two used mixed qualitative and quantitative methods (combining interviews, focus groups or stakeholder workshops with surveys^34^ ^41^); and one used audit.^33^

### What is known about the barriers and facilitators to good communication?

The themes that were developed to describe the barriers and facilitators to good communication are presented below.

### Barriers to good communication

#### A lack of continuity of care

A lack of informational and relational continuity of care was consistently raised as a barrier to communication. Findings related to this theme were extracted from fifteen included articles.^2426^,^28 30^

Patients were often reluctant to access care OOH because they did not want to seek ‘advice from a stranger’ when they were scared and vulnerable.^24 31 38^ Compared with healthcare professionals accessible during the day who could make them feel ‘known’ and ‘listened to’, levels of trust in OOH professionals were much lower. Some patients even delayed seeking help so that they could see their own healthcare team.^24 30^ OOH professionals sympathised with this perspective, finding it difficult to build trust as they entered patients’ lives for a ‘snapshot’ of their palliative care journey.^28 36^ They could not offer to personally follow up with patients to provide reassurance, as they were unlikely to be on shift when follow-up was required.^37^ Some struggled to be completely honest because they had not built up familiarity and were uncomfortable giving bad news as a stranger.^28 36^

OOH professionals rarely had access to full patient notes, leading to frustrations for patients and families^24 26 27 32^ and challenging discussions for professionals.^32 33 38 39^ Patients and families expressed dissatisfaction about OOH professionals’ lack of knowledge about their situation, sometimes having to repeat the same information several times in one care episode (eg, over the phone then in person). This caused delays to care, often when patients were in pain and desperate to receive help. Patients and family members were worried about being relied on to provide accurate information about their medical history and medications.^27 30 32 40^ Professionals also had to ascertain their levels of awareness of their prognosis, and their preferences regarding treatment escalation.^32 36 38 39^ They felt that the absence of informational continuity made them look ill-informed and exacerbated trust issues:

To go in blind as is done on various different occasions with no notes on a patient is absolutely crucifying, it’ s horriﬁc, you look so stupid.^39^

#### Remote consultations

12 papers^2527 29 30 32 35^,^41^ identified barriers to good communication created by the reliance on remote consultations and the isolation of working within the OOH system.

There were issues with communication between different people working within the OOH system.^27 32 37 38 40^ Patients reported receiving conflicting information from different healthcare professionals across the same care episode, and triagists expressed frustration when their advice was not followed by GPs conducting home visits:

the frustrating part is when, there is some other professional colleagues, they don’ t always understand, again I can cite an example only recently, where I felt at the end of the phone that this man could have done with a dose of haloperidol to calm him down. You know, while what the GP did, who visited, he ignored my advice he actually increased the dose of diamorphine.^37^

OOH professionals, patients and families all raised difficulties with obtaining or conveying information during remote contacts. Even where information was available, relying on notes could lead to out-of-date information and misunderstandings.^35 38 40 41^ Working OOH meant professionals could not clarify information with other healthcare professionals. GPs working OOH were often uncomfortable making decisions over the phone without having seen patients, often only receiving third-hand information through district nurses, care home staff or family members.^3035^,^37 39 40^ This could lead to conflict with nurses when, rather than wanting advice, they were seeking a signature for a prescription, for instance. Some GPs felt that terminal care patients should always be seen in person:

I think that this is awful, making such important decisions about the medication of a terminal patient by telephone. My opinion is that you have to visit the patient personally.^35^

Patients and families described the ‘rigmarole’ of getting an OOH appointment or visit, having to go through lengthy questioning and sometimes feeling the process was hostile.^24 25 27 29 30 32 38^ This was made more difficult for family members who were trying to convey information on behalf of the patient:

I just had all this hassle … and I was reduced to tears. If you could get a bit more help when you phone without all the questionnaire things you know.^38^

#### Delegitimising help seeking

Four papers included examples of worries or experiences of the legitimacy of help-seeking being questioned.^26 27 30 31^ Patients expressed worries about wasting doctors’ time OOH^27 31^:

I didn’ t want to be a nuisance… I thought I maybe, it wasn’ t that serious, you know, there’ s probably somebody more serious than I.^31^

Communication once patients did seek help was therefore particularly important to ensure they did not delay contacting the service in the future. However, several studies found evidence of patients and families feeling that their problems were delegitimised and that the healthcare professional should not have been called out for the problem^26 27 30^:

Here am I, a GP administering an injection. Nurses can do that. You don’ t need a doctor to do this. And I think the point perhaps he was really saying is that there may be some serious doctory things going on that he ought to be attending to.^27^

#### Specialist work in a generalist system

10 papers found evidence that the specialist work required in palliative care was sometimes incompatible with OOH systems.^2425 30 34^,^38 40 41^ The challenge was summarised by one GP who “*found it difficult to bring together the ethos of OOH and that of palliative medicine; one being an acute, fast-paced service, the other requiring time and listening skills”*.^36^ Professionals felt pressure to get through consultations quickly, meaning that sensitive communication and emotional investment were difficult^30 36 37^:

you’ re extremely busy and have a whole lot of other calls stacked up, so we lack time to sit down and give people the time to explain what is going on in a way that is not hurried.^30^

Some professionals lacked confidence having these conversations due to limited palliative care expertise, not having adequate information about the patient and the perception that patients had low expectations of OOH professionals.

you may have more of a thing to prove yourself, don’ t know if that’ s the right word, but you want them to have conﬁdence in you… They assume they’ ll get a bad service and be fobbed oﬀ.^36^

This was a reasonable concern as several papers reported negative perceptions of OOH GPs from patients and other healthcare professionals^24 25 27 34 38 40 41^:

I just feel that there should be somebody available even if it’ s only on the end of a phone that you can talk to and be confident that they know what you’ re asking… somebody that is used to dealing with that medication… how many locums and GPs have that knowledge?^24^

Patients and other healthcare staff, such as care home workers, expressed concerns that this lack of confidence in these interactions sometimes led to inappropriate hospital admissions because they are “*too scared to deal with that patient”*^30^ or “*wary of conservative management.”*^41^

### Facilitators to good communication

#### Access to patient notes and handover documents

Most barriers involved a lack of access to information about patients. It therefore follows that care summaries and handover notes from in-hours services could help facilitate communication, as shown in eleven papers.^2226 27 29^,^32 34 36 39 41^ There were different approaches to information sharing, but it often involved a summary document informing OOH professionals about patients’ medical history, medications and advance care planning. These were welcomed by OOH professionals as an aid to communication and rapport building^30^,^3235^:

you know the patient is fully aware, relatives aware of prognosis and outcome, … and you know their wishes… it saves you putting your foot in it.^32^

This was particularly helpful when in-hours healthcare professionals had made clear plans with patients so that they did not have to have these conversations with ‘strangers’ in an urgent, OOH setting.^36 39^ The remote nature of OOH services meant that accessing electronic records was sometimes difficult, and so several papers suggested that notes should be left in patients’ homes.^3336^,^38 41^

Patients appreciated professionals appearing well informed about their history,^25 26 39^ helping them to feel *‘known’*.^32^ There was no evidence of patient concerns about sharing notes,^24 32 38 39^ although some GPs raised concerns about confidentiality and informed consent.^39^ Patients and families were particularly pleased when handover documents and special notes triggered fast-tracking of their case, reducing the ‘rigmarole’ of accessing care.^30 31 38^ Those who had negative experiences thought handover notes would improve communication:

I’ m sure [ handover forms ] would be helpful because at least they could know a little bit of background about you…you’ d think that, in this day and age, with the computers, that you’ d be able to put in a number of some sort and be able to just get what you want there.^32^

#### OOH professionals’ manner

Seven papers described the importance of the OOH professional’s manner during contacts.^25^,^2729^ Patients and families often understood the challenges of the OOH system. The way they were treated by OOH professionals was what impacted their views of the service provision and communication:

Well I don’ t see how they can ever cover, having everybody’ s information who happens to phone up that particular night can they? So I mean that’ s got to be too difﬁcult. What you really want them to do, is to show some sort of understanding and try to give you some advice and help least you feel as if someone has now got your case in hand and you won’ t be left to wander the moors.^27^

Features of healthcare professionals’ communication that were consistently reported as being valued were honesty, empathy, confidence, listening and clearly explaining options.^25^,^2729 30 35^ It was important they treated the patient as an individual and listened to family carers, recognising their unique expertise.

Reassurance that their concerns were legitimate was important, both before and during consultations.^27 30^ It was helpful when in-hours healthcare professionals gave clear information about who to contact and when. This may be particularly important for those patients without family support, as it was often family members who contacted the OOH service and felt more empowered to do so.^27 29 31^ They were more positive about their experience if OOH professionals reassured them they had done the right thing:

At first I used to get myself in a state—what a shame bringing them out… I was very much reassured by them when I apologised, they said ‘ no apologies, that’ s why we’ re here’ .^30^

Willingness to visit patients at home rather than over the phone was appreciated by patients and families and raised by professionals as important for safety and reassurance.

Time is not the most important thing. Being there is important. Often it sufﬁcient just because you’ ve been there, and then they can carry on.^35^

#### Support from colleagues

Eight papers^2529 33^,^37 41^ suggested that OOH professionals working closely with other professionals reduced isolation and improved confidence. Improved professional-to-professional communication had knock-on effects for patients and families who appreciated when different parts of the OOH system cooperated to provide coordinated care.^29^

A key collaborative relationship was between OOH professionals and specialist palliative care professionals.^33 36 37 41^ This included informal conversations and professional-to-professional advice lines. Some OOH GPs showed reluctance to request advice or were not aware of who to call, mirroring patients’ barriers. Those who had sought specialist advice were positive about their experiences of this communication:

generally I find that Oncology and Palliative Care docs do seem to be quite sympathetic to GPs… I think they realise that we sometimes probably struggle with some of these things.^37^

Similar to the findings for patients therefore, OOH professionals needed to know who to call and feel supported when they did call. Some reported that access to specialist advice increased their confidence dealing with issues in the home, reducing unnecessary hospital admissions.

Another key relationship was with healthcare professionals working in the community, including district nurses and nursing or care home staff.^34 37^ While some OOH GPs were uncomfortable relying on nurses’ knowledge, others found this useful and their decision-making could be led by nurses:

I have had some very positive experiences as well, where the nurses have been very useful. And that’ s been like a hand-holding thing, whilst I’ ve been on-call for OOH.^37^

Trust in this relationship was vital for good communication. One study found that the Gold Standards Framework for Care Homes improved trust and collaborative working by *“providing nursing homes with frameworks for considering end-of-life care, relevant training, networking and support.”*^34^

## Discussion

### Key findings and implications

This scoping review has identified 18, mostly qualitative, papers with findings about communication during OOH contacts with primary care services for people with a terminal illness. We have synthesised the barriers and facilitators to good communication in this setting that have been identified across the literature.

Limited availability of information about the patient and the absence of existing relationships led to patients and families having important, sensitive conversations with professionals whom they sometimes perceived to be ill-informed strangers. This was exacerbated by the need for remote contacts involving repeating information to different OOH professionals. The literature suggests that OOH services are designed around acute problems and are therefore not well-suited to the careful, skilled communication often required in palliative and end-of-life care. Despite these challenges, there were examples of good communication, where access to handover notes and support from colleagues improved experiences for professionals and patients. Patients often understood the challenges of working OOH, and having empathetic, reassuring professionals could overcome these difficulties.

The first implication of these findings is the need for access to patient notes OOH. This fits with the Department of Health’s 2016 recommendation of a national rollout of electronic palliative care coordination systems (EPaCCS) by 2020^42^ and the Gold Standards Framework requirement that palliative care patients should be identified in-hours and handover notes provided to OOH services.^43^ There is evidence that EPaCCS can reduce hospital admissions and are beneficial to OOH professionals, but some healthcare professionals working in-hours view their completion as burdensome, hindering their implementation in practice.^39 44 45^ In England, studies have shown low rates of EPaCCS and anticipatory care planning in records of deceased patients.^46 47^ Lessons could be learnt from Scotland where 69% of people who died had a Key Information Summary (KIS; a type of EPaCCS) and these were initiated earlier in the disease trajectory.^48^ The authors highlight national incentives and triggers to complete KIS, such as admission to nursing/care homes, as supporting the rollout.^48^

The second implication of the findings is the need for patients and families to be able to access OOH healthcare professionals skilled in palliative care communication. There are examples of specialist services operating OOH, which have been successful in improving experiences of patients and families and reducing hospitalisations.^49 50^ OOH specialist palliative care services should be available more widely, but even with these in place, OOH professionals are likely to see some palliative or end-of-life patients. They therefore need training and support to provide these services. A Marie Curie workshop for palliative care professionals and user representatives recommended embedding palliative care nurses in community OOH teams, training to upskill generalists working OOH, and providing specialist professional-to-professional advice lines OOH.^51^ Collaborating with palliative care professionals may be preferable to formal training to upskill generalists and improve their confidence.^52^

### Strengths and limitations

Strengths of this scoping review include the systematic approach to search, selection and data extraction procedures. While ideally we would have sought PPI input at an earlier stage, sharing initial findings with PPI representatives aided interpretation of the findings. A limitation was that searches were limited to English-language papers due to a lack of translation resources. Communication practices may differ across languages and cultures, which this review has not captured.

Only one included paper was published in the last 5 years. Changes to practices around data sharing and ways of working may therefore have addressed some issues raised in the review, and COVID-19 may have changed the landscape of remote consulting by normalising phone consultations. However, the report published by Marie Curie in 2022^51^ raised many of the same issues. Similar issues have been reported in the literature before changes to GP OOH contracts, suggesting these challenges are enduring.^24 27^

### Future research

Only one paper had a stated aim specifically about communication during OOH contacts for people with a terminal illness.^37^ More research is therefore needed to directly address the research questions of this review. The majority of studies used interviews or focus groups, which provided insights into participants’ experiences. However, communication about dying is multidirectional between professionals, patients and families.^9^ To capture this and characterise common communication practices and their impact on patients and family members, research using observations and/or recordings of consultations is needed.

## Conclusions

This scoping review has synthesised existing literature about communication during OOH contacts with primary care services for people with a terminal illness. Research specifically focusing on common communication practices and their impact on patients and families is needed. The experiences of patients and families, and outcomes of OOH contacts, could be improved through consistent availability of patient notes, palliative care training for OOH professionals and access to support from palliative care colleagues.

## Supplementary material

10.1136/bmjopen-2025-105738 online supplemental file 1

10.1136/bmjopen-2025-105738 online supplemental file 2

## Data Availability

Data sharing is not applicable as no datasets were generated and/or analysed for this study.
