# Supplementary material for: Communication during out-of-hours primary care contacts for people with a terminal illness: a scoping review
Source: BMJ Open. 2026 Feb 27;16(2):e105738. doi: 10.1136/bmjopen-2025-105738 (PMC12959005; doi:10.1136/bmjopen-2025-105738)
Supplement: online supplemental file 1 [file bmjopen-16-2-s001.docx]

**Appendix A: Full Search strategy**

**MEDLINE (Ovid)**

| 1. exp Terminal Care/ |
| --- |
| 1. exp Palliative Care/ |
| 1. ("end of life" or "end-of-life" or palliative or terminal* or dying or die or died or bereav* or hospice* or "palliative service*" or "advance care plan*").ti,ab,kf. |
| 1. 1 or 2 or 3 |
| 1. exp Communication/ |
| 1. exp communication barriers/ |
| 1. exp Interpersonal relations/ |
| 1. (communicat* or talk* or discuss* or "advance care plan*" or conversation* or interact* or decision-making or "decision making").ti,ab,kf. |
| 1. 5 or 6 or 7 or 8 |
| 1. ("out of hours" or "out-of-hours" or "after hours" or evening? or night? or weekend? or "urgent care" or unscheduled or "telephone triage").ti,ab,kf. |
| 1. 4 and 9 and 10 |
| 1. limit 11 to english language |

**EMBASE (Ovid)**

| 1. exp Terminal Care/ |
| --- |
| 1. exp Palliative therapy/ |
| 1. ("end of life" or "end-of-life" or palliative or "terminally ill" or "terminal illness" or dying or die or died or bereav* or hospice* or "palliative service*" or "advance care plan*").ti,ab,kf. |
| 1. 1 or 2 or 3 |
| 1. exp interpersonal communication/ |
| 1. exp interdisciplinary communication/ |
| 1. exp professional-patient relationship/ |
| 1. (communicat* or talk* or discuss* or "advance care plan*" or conversation* or interact* or decision-making or "decision making").ti,ab,kf. |
| 1. 5 or 6 or 7 or 8 |
| 1. ("out of hours" or "out-of-hours" or "after hours" or evening? or weekend? or "urgent care" or unscheduled or "telephone triage").ti,ab,kf. |
| 1. 4 and 9 and 10 |
| 1. limit 11 to english language |

**PsycInfo (Ovid)**

| 1. exp Terminally ill patients/ |
| --- |
| 1. exp Palliative Care/ |
| 1. ("end of life" or "end-of-life" or palliative or terminal* or dying or die or died or bereav* or hospice* or "palliative service*" or "advance care plan*").ti,ab,id. |
| 1. 1 or 2 or 3 |
| 1. exp Communication/ |
| 1. (communicat* or talk* or discuss* or "advance care plan*" or conversation* or interact* or consultation* or decision-making or "decision making").ti,ab,id. |
| 1. 5 or 6 |
| 1. ("out of hours" or "out-of-hours" or "after hours" or evening? Or night? or weekend? or "urgent care" or unscheduled or "telephone triage" or telecare or hotline?).ti,ab,id. |
| 1. 4 and 7 and 8 |
| 1. limit 11 to english language |

**CINAHL (Ebsco)**

| 1. (MH “Terminal Care+) OR (MH “Terminally Ill Patients+”) OR (MH “Palliative Medicine”) |
| --- |
| 1. (TI "end of life") OR (TI "end-of-life") OR (TI palliative) OR (TI terminal*) OR (TI dying) OR (TI die) OR (TI died) OR (TI bereav*) OR (TI hospice*) OR (TI "palliative service*") OR (TI "advance care plan*") OR (AB "end of life") OR (AB "end-of-life") OR (AB palliative) OR (AB terminal*) OR (AB dying) OR (AB die) OR (AB died) OR (AB bereav*) OR (AB hospice*) OR (AB "palliative service*") OR (AB "advance care plan*") |
| 1. S1 or S2 |
| 1. (MH Communication+) |
| 1. (MH “Professional-Patient Relations+”) OR (MH “Interprofessional Relations+”) OR (MH “Professional-Family Relations”) OR (MH “Patient-Family Relations”) |
| 1. (TI communicat*) OR (TI talk*) OR (TI discuss*) OR (TI "advance care plan*") OR (TI conversation*) OR (TI interact*) OR (TI decision-making) OR (TI "decision making") OR (AB communicat*) OR (AB talk*) OR (AB discuss*) OR (AB "advance care plan*") OR (AB conversation*) OR (AB interact*) OR (AB decision-making) OR (AB "decision making") |
| 1. S4 OR S5 OR S6 |
| 1. (TI "out of hours") OR (TI "out-of-hours") OR (TI "after hours") OR (TI evening*) OR (TI night*) OR (TI weekend*) OR (TI "urgent care") OR (TI “unscheduled care” )OR (TI "telephone triage") OR (AB "out of hours") OR (AB "out-of-hours") OR (AB "after hours") OR (AB evening*) OR (AB night*) OR (AB weekend*) OR (AB "urgent care") OR (AB “unscheduled care” )OR (AB "telephone triage") |
| 1. S3 and S7 and S8 |

**Grey Literature**

Websites: EAPC, EurOOHnet, Marie Curie, Macmillan, National Voices

ProQuest search terms: ((summary("terminal care" OR "palliative care" OR "end of life" OR "end-of-life" OR dying OR hospice) AND summary(communicat* OR discuss* OR "advance care plan*" OR conversation* OR interacti*) AND summary("out of hours" OR "out-of-hours" OR "after hours" OR evening? OR night? OR weekend? OR "urgent care" OR unscheduled)) NOT stype.exact("Scholarly Journals" OR "Newspapers" OR "Trade Journals" OR "Historical Newspapers" OR "Books" OR "Conference Papers & Proceedings" OR "Reports")) AND (la.exact("ENG") NOT stype.exact("Magazines" OR "Historical Periodicals" OR "Wire Feeds"))
