# Supplementary material for: Communication during out-of-hours primary care contacts for people with a terminal illness: a scoping review
Source: BMJ Open. 2026 Feb 27;16(2):e105738. doi: 10.1136/bmjopen-2025-105738 (PMC12959005; doi:10.1136/bmjopen-2025-105738)
Supplement: online supplemental file 2 [file bmjopen-16-2-s002.docx]

**Appendix B: Detailed summary table of included papers**

| **Author (date)** | **Country** | **Stated research question/aim** | **Population** | **Focus of relevant evidence*** | **Methods*** | **Relevant findings: Barriers and facilitators to good communication** |
| --- | --- | --- | --- | --- | --- | --- |
| Adam et al. (2015)^31^ | Scotland | To explore the experiences, views, and opinions of patients and their caregivers who have used OOH services for help with managing cancer pain | 11 cancer patients 4 caregivers | Communication between professionals and patients/families | Semi-structured interviews | **Barriers:**  Patients didn't want to be a nuisance and didn't always know who to contact/how  'Rigmarole' of repeatedly telling story, especially while in pain  **Facilitators:**  Appreciation of informational continuity of palliative care summaries. Also meant patients could skip triage  Caregivers empowered patients to seek help  Specific instructions of who/how to contact OOH  Satisfaction often related to manner of the person they spoke to  It wasn’t as important OOH to see the same professional as it was in hours, patients just wanted their pain treated |
| Asprey et al. (2013)^32^ | England | To work with service users and providers to optimise the design and implementation of handover forms to support the transfer of information between daytime and OOH primary care services for patients with palliative care needs | 8 patients/carers  15 GPs (mix of in and OOH) 13 nurses (mix of in and OOH) 1 administrator | Communication between professionals and patients/families | Action research: Interviews and focus groups (stakeholder feedback also gathered but did not contribute to findings about communication) | **Barriers:**  Having to repeat information to OOH doctors. This could be exhausting when unwell. OOH doctors felt it was intrusive to keep asking for the same information. Families often don't know details so OOH doctors have to ask a lot of questions  Lack of consistency in amount/quality of information in handover forms. Information could be out of date  Lack of online access to notes in mobile teams meant messages being relayed over the phone and sometimes missed  **Facilitators:**  Patients/carers would be happy to share their notes (and expected this to already be happening). OOH doctors felt handover forms allowed them to be more sensitive and enabled better decision making |
| Badger et al. (2012)^34^ | England | To evaluate the impact of a training programme to improve end-of-life care in nursing homes, on collaboration between nursing home staff and other health practitioners | Survey: 79 nursing homes (49 completed both pre and post surveys) Interviews/focus groups: 14 care home managers 26 Registered Nurses 30 care assistants 4 domestic staff 1 primary care liaison nurse | Communication between different professionals | Mixed methods: pre- and post- programme surveys and case studies using interviews and focus groups | **Barriers:**  Difficulties accessing GPs OOH  Staff felt that GPs not being familiar with patients/patient group and OOH service not listening to care home staff led to inappropriate transfers to hospital  Relationships with OOH were resistant to change - they have less of a relationship with homes and don't know their culture  **Facilitators:**  Improved communication and collaborative working OOH services was reported post GSFCH implementation. Trust and confidence were increased by providing a framework for training, support and networking. Managers thought improved communication with OOH GPs reduced unnecessary hospital admissions. |
| Fergus et al. (2010)^38^ | Scotland | To identify key issues relating to OOH care for palliative care patients from patient, carer and professional perspectives, and integrate their suggestions for improvement | Interviews: 6 patients 1 carer 17 GPs providing in and OOH care 5 district nurses 2 palliative care physicians 5 NHS24 nurse advisors | Communication between different professionals  Communication between professionals and patients/families | Rapid participatory appraisal: interviews and observations | **Barriers:**  Out of date or ambiguous in hours notes on patients can cause problems/confusion. Inadequate information led to time spent in consultations finding out details (including patient's understanding of their illness). Lack of information about involvement of specialist palliative care could lead to inappropriate admissions. No reliable system in place to record the outcome of each visit for the next visiting professional.  Requirement to go through NHS24 to speak to a doctor was frustrating. Patients found NHS24 a 'rigmarole', especially asking lots of questions of an ill person. NHS24 nurses couldn't access information about earlier calls/visits.  Patient/carer reluctance to call NHS24 and speak to someone they don't know  OOH GP unaware of professional palliative care advice lines they can call  **Facilitators:**  Patients were happy for notes to be shared with OOH. Patient-held notes were available DN were involved, this was useful.  Providing ways to contact professionals directly rather than going through NHS24 would be useful |
| Hall et al. (2012)^39^ | Scotland | To identify key issues related to the introduction of ePCS from primary care and OOH staff, to identify facilitators and barriers to their use, to explore the experiences of patients and carers and to make recommendations for improvements. | 12 GPs (7 with OOH involvement) 3 practice nurses 1 practice manager 2 patients 4 carers | Communication between different professionals  Communication between professionals and patients/families | Semi-structured interviews | **Barriers:**  GPs worried about consent for notes sharing for people with dementia.  Underutilisation of forms (especially for non-cancer patients) was a problem and meant OOH staff didn't always look for it  **Facilitators:**  Patients and carers were not worried about confidentiality around notes sharing  Clinicians found patients like that ePCS means they don't have to retell their story in OOH (including things like DNAR). OOH doctors found it avoided communication problems due to having to work out patients/carers awareness/feelings about prognosis etc. |
| Hanratty et al. (2014)^40^ | England | To understand the experiences, influences and consequences of transitions between settings for older adults at the end of life | 30 older patients in their last year of life  118 bereaved caregivers  43 providers and commissioners of services in primary care, hospital, hospice, social care and ambulance services. | Communication between different professionals  Communication between professionals and patients/families | Interviews (Routine data also used but did not contribute to findings on communication) | **Barriers:**  Notes from in hours GP's can be vague (e.g. 'no heroics')  Incompatible IT systems caused problems  Family carers worried OOH calls would take too long and end in hospital admission  Specialists and hospital doctors raised issues of OOH doctors assessing over the phone/not knowing the person resulting in inappropriate admissions  Family carers had to relay information- they worried about those without close family to do this |
| King et al. (2004)^26^ | England | To examine family carers’ experiences of OOH community palliative care, with a particular emphasis on the services provided by GP and district nurse OOH services | 15 bereaved carers | Communication between professionals and patients/families | Semi-structured interviews | **Barriers:**  Poor communication about who to call and when was confusing. Poor communication about when to expect clinician to arrive led to anger  Failure to listen to the carer  Frustrating having to go through information repeatedly  Less warmth when describing OOH GPs than in hours/ district nurses- lack of familiarity  **Facilitators:**  Pleased when OOH GP was well informed about patient- this makes it easier to build rapport as professionals don't have to spend time eliciting the patient's history  Appreciated when in hours GPs visited out of hours |
| Leydon et al. (2013)^24^ | England | 1. To understand the experiences of palliative care patients when accessing or making decisions about OOH services 2. To illuminate barriers and enablers to accessing appropriate and timely care following the introduction of the 2004 New General Medical Services Contract | 22 patients (a further 10 were recruited but not interviewed) 4 carers (2 current, 2 bereaved) | Communication between professionals and patients/families | Prospective interviews | **Barriers:**  Patients found it hard not having a familiar person who knew their story to speak with. Not being known meant having to retell their story and made communication and decision making difficult  Some participants described not seeking OOH care and waiting to see their own GP  **Facilitators:**  Authors suggest 'informational continuity' (e.g. from better communication between in and out of hours services) could reduce feelings of not being known  Feelings of 'being known' were associated with being listened to |
| Lloyd-Williams & Rashid (2003)^33^ | England | Pilot an OOH palliative care advice line for professionals and audit the content of calls in the first year | 98 calls from: OOH GPs (55%) Community nurses (34%) Junior hospital doctors (10%) | Communication between different professionals | Audit | **Barriers:**  Professionals had no background information on patients  **Facilitators:**  The advice line prevented some transfers to hospital  Suggest patient-held notes to allow better communication  The advice line was used appropriately suggesting fears of overuse are unfounded |
| Mason et al. (2022)^29^ | Scotland | To document the frequency and patterns of use of unscheduled healthcare by people in their last year of life and understand the experiences and perspectives of patients, families and professionals about accessing unscheduled care OOH. | 50 patients/carers (current and bereaved) 8 GPs | Communication between professionals and patients/families | Prospective interviews and focus groups (routine data also used but did not contribute to findings on communication) | **Barriers:**  Carers frustrated by having to prove they are next of kin before getting to explain the problem. They viewed NHS24 as having lots of questions and longs waits (even if they hadn't used it)  **Facilitators:**  Coordination between services to avoid hospital admission  Clear explanation about admission, rather than attempting to influence the decision |
| Richards et al. (2011)^27^ | England | To explore the experiences of people with advanced cancer and/or their caregivers accessing OOH care | 13 advanced cancer patients 15 caregivers | Communication between professionals and patients/families | Semi-structured interviews | **Barriers:**  Some felt dismissed by OOH doctor, others delayed calling as they didn't want to bother doctors if it wasn't serious enough  One caregiver said doctor wouldn't acknowledge her fear of her father dying  Frustration at having to repeat information (even in repeat phone calls the same evening) when the patient is in pain. Worried about reliance on patients/caregivers to remember information. Lack of continuity/clear communication between different professionals during same episode of care (e.g. between different doctors or doctors and ambulance service)  Understood wouldn't be able to see someone who knew them, but didn't understand why couldn't share notes  **Facilitators:**  Positive experiences recounted in less detail but included being impressed by doctor's manner and follow up; quick responses; and feeling professionals are 'tuned in' and experienced. They wanted doctor to show understanding/support even if they didn't have all the information.  Knowledge and reassurance identified as most important factors  Caregiver often called/persuaded patient to call |
| Schweitzer et al. (2011)^35^ | Netherlands | 1. How do GPs assess the quality of OOH palliative care provided by GP co-operatives in the Netherlands? 2. Which factors contribute to a better or worse quality of OOH palliative care according to the GPs? 3. Which improvements in the quality of OOH palliative care could be made according to the GPs, and how can these be achieved? | 20 GPs working OOH | Communication between different professionals  Communication between professionals and patients/families | Focus groups | **Barriers:**  Felt that problems that require a home visit are often dealt with over the phone  Concerns that handover information that includes e.g. DNAR could be misconstrued and lead to patients being neglected  OOH service is designed around acute problems meaning there isn’t time for the complex discussions that are needed  Confusing communication with care home staff who don't know the patient well  **Facilitators:**  Family are a useful source of information when notes are missing  Helpful if usual GP has provided info/is contactable: Patients appreciate feeling the doctor knows them, doctors know what to expect  Home visits give more moral support than phone call |
| Seamark et al. (2014)^25^ | England | To elicit family carers’ views about the community support that made death at home possible | 59 bereaved carers | Communication between professionals and patients/families | Semi-structured interviews | **Barriers:**  Frustrated by delays out of hours and reluctant to call  **Facilitators:**  Appreciated availability of own GP  Some were impressed by professionalism and information that emergency doctor had  Some were comforted by the ease with which they could contact someone OOH |
| Taubert & Nelson (2010)^36^ † | Wales | To explore factors influencing GP's confidence in dealing with symptom control and palliative care provision outside regular working hours | 9 OOH GPs | Communication between different professionals  Communication between professionals and patients/families | Semi-structured interviews | **Barriers:**  Needs of the OOH service to see patients quickly prevents the required sensitivity and time within consultations. This could be distressing for doctors  Doesn't feel right for sensitive conversations (e.g. prognosis) to come from a 'stranger'. More challenging to be honest/frank with patients when you don't know them  Feel patients often expecting a bad service/to be fobbed off so start on a bad footing  Not speaking to other professionals face-to-face is difficult. Asked to make changes to meds without seeing the patient, doesn't feel safe  Can be difficult to access specialist advice OOH (or lack of knowledge about who/how to contact specialist services)  **Facilitators:**  Written notes in patient's homes and electronic handover forms would be helpful |
| Taubert & Nelson (2010)^37^ † | Wales | To explore factors that OOH GPs identified as detrimental or beneficial for good communication between themselves, patients, relatives and other professionals, specifically to palliative care encounters | 9 OOH GPs | Communication between different professionals  Communication between professionals and patients/families | Semi-structured interviews | **Barriers:**  Only get a 'snapshot' of the person. Can't follow-up with the patient  Lack of handover from in-hours causes confusion and leaves decision making to OOH  Lack of knowledge of specialist advice lines GP could call  Advice from GP on clinician call often ignored at home visit  Can feel unsafe to makes decisions over the phone without seeing the person. Some didn't want to go on what a nurse says and (e.g.) sign a prescription, can cause conflict with nurses. Can feel alone, unsure who to call  **Facilitators:**  Notes in patients' homes would be helpful  Being able to speak to PC specialists helps confidence with plan  Work well with district nurses |
| Taubert et al. (2011)^28^ † | Wales | To determine, by interviewing GPs who provide OOH care, aspects of care provision that augmented or challenged palliative care delivery | 9 OOH GPs | Communication between professionals and patients/families | Semi-structured interviews | **Barriers:**  Lack of existing doctor-patient relationship makes it harder to put people at ease and be honest. More formalities and lowered trust and confidence  It is not possible to learn from experiences as GPs do not get to find out the outcome of the consultation |
| Thomas (2009)^41^ | England | What helps to avoid hospital admission of patients who want to die at home when a crisis occurs in the OOH period? | Stakeholder workshops: 20 practitioners/ managers (In hours GP, OOH GP, London Ambulance Service, hospital) 1 patient  Surveys: 24 GP practice managers 15 community nurses | Communication between different professionals  Communication between professionals and patients/families | Whole system participatory action research using stakeholder workshops and a survey | **Barriers:**  OOH can appear to not be listening and unaware of history. GPs say this is because of lack of access to notes/notes not providing all of the information that is needed  Workshop attendees agreed on barriers to good communication as: 1. Emotional difficulties dealing with 'conservative management' 2. Difficulties integrating care across systems 3. Issues getting consent for note sharing  **Facilitators:**  Suggest a records at home system and other communication systems to allow sharing of notes between professionals |
| Worth et al. (2006)^30^ | Scotland | To explore the experiences and perceptions of OOH care of patients with advanced cancer, and with their informal and professional carers | 39 advanced cancer patients  67 informal carers  50 professionals (the patient’s GP, district nurses, out-of-hours service personnel, palliative care specialists and hospital staff) | Communication between professionals and patients/families | In-depth interviews and focus groups | **Barriers:**  Reluctant to seek help. Some felt doctor didn't think it was a legitimate reason to come out. Some felt call handlers were unsympathetic and that they had to justify themselves. Negative experiences of abrupt responses/ reluctance to visit made patients/carers not want to use service  Many patients preferred to wait until morning to speak to their own GP or specialist nurse, instead of using an 'impersonal service'  Didn't think it was worth giving whole story over the phone when would have to do so again in person  Worries about impersonal service or doctors not listening and sending to hospital (partly based on hearsay/media reports)  Doctors thought there was a lack of continuity and a rushed service; lack of time to talk properly with families to ensure right decisions are made  **Facilitators:**  Carers often called on patient's behalf (more determined to get someone to see the patient). Encouragement from doctors that it was the right decision to call helped  Own practice sending info to OOH meant fast-tracking and so less waiting and less repeating story. Good communication with in hours doctors allows for better decision making  Good communication, confidence and empathy more important than seeing the same person. Need for reassurance, comfort and advice |

*Only methods used to collect relevant evidence included

†Papers based on the same dataset
