# Supplementary material for: Communication during out-of-hours primary care contacts for people with a terminal illness: a scoping review
Source: BMJ Open. 2026 Feb 27;16(2):e105738. doi: 10.1136/bmjopen-2025-105738 (PMC12959005; doi:10.1136/bmjopen-2025-105738)
Supplement: Supplementary file 3 [file bmjopen-2025-105738.reviewer_comments.pdf]

PEER REVIEW HISTORY

BMJ Open publishes all reviews undertaken for accepted manuscripts. Reviewers are asked to complete a checklist review form and are provided with free text boxes to elaborate on their assessment. These free text comments are reproduced below.

ARTICLE DETAILS

Title (Provisional)

Communication during out of hours primary care contacts for people with a terminal illness: A scoping review

Authors

Anderson-Kittow, Rebecca; Dillon, Roisin; Barnes, Rebecca K

VERSION 1 - REVIEW

**Reviewer**	**1**
**Name**	**Pocock, Lucy**
**Affiliation**	**University of Bristol, Centre for Academic Primary Care**
**Date**	**08-Jul-2025**
**COI**	**None**

Many thanks for the opportunity to review this paper, which is a scoping review exploring what is known about communication during contacts between people with a terminal illness and OOH primary care.

The introduction and methods are well-written and clear.

On page 4, there is a typo in the subheading, which should read: Eligibility criteria, study sel

ction and data extraction

In the results section, although this information is presented in table 1, I think it would be helpful to state in the text the outcomes relating to these research questions:

Which participant groups have been included (i.e. patients, families, healthcare professionals)?

Which methods have been used to investigate what happens?

The results are clear and resonate with my professional experience.

The discussion and conclusion provide a coherent summary of the results and implications of the findings.

**Reviewer**	**2**
**Name**	**Heckel, Maria**
**Affiliation**	**Friedrich-Alexander-Universität Erlangen-Nürnberg, University Hospital Erlangen, Department of Palliative Medicine**
**Date**	**15-Dec-2025**
**COI**	**None**

Thank you very much for your valuable work on this important issue of day-to-day care. It is crucial to gain more knowledge on the barriers and facilitators of communication during out-of-hours primary care contacts for people with a terminal illness. The review was conducted and reported in a scientifically accurate and careful manner. The tables and graphs are easy to read and summarize procedures and results well. The narrative results section is highly descriptive.

I have some minor comments:

There are some typos (introduction page 2 line 49-50).

The introduction does not explain why the focus is on primary care, as reflected in the inclusion criteria. The first paragraph emphasises pain control as a major issue in palliative care. While this may be one of the most frequent problems, other symptoms could also be mentioned.

In the methods section you mention that "Following the JBI guidance, only concepts and findings relevant to the research questions were extracted." Please specify the concepts relevant to the research questions.

I appreciate the PPI very much. I recognizse that it is quite difficult to describe the impact. However, could you provide more detail about the feedback received and how it helped with the interpretation of the findings?

VERSION 1 - AUTHOR RESPONSE

Response to Reviewer 1, Dr. Lucy Pocock:
Thank you for your positive and useful comments. Please find our responses in the table below:

Comment	Response
The introduction and methods are well-written and clear.	Thank you
On page 4, there is a typo in the subheading, which should read: Eligibility criteria, study sel**e**ction and data extraction	Typo corrected
In the results section, although this information is presented in table 1, I think it	The results have now been split into the three questions stated in the introduction. The

would be helpful to state in the text the outcomes relating to these research questions:<br>Which participant groups have been included (i.e. patients, families, healthcare professionals)?<br>Which methods have been used to investigate what happens?	section about which participant groups have been included now reads: "A total of 124 patients, 287 family members/informal carers, a further 58 patients/carers (not reported separately in the papers) and 485 professionals (including GP, nurses, practice managers, care staff, palliative care specialists, commissioners and administrators) participated in the included studies. Nine papers included findings about communication between professionals and patients/families, four with patients/families only as participants[24–27], one with professionals only[28], and four with both patients/families and professionals[29–32]. Two papers included findings on communication between different professionals, both with professionals only as participants[33,34]. Seven papers covered both communication between professionals and patients/families and communication between different professionals, three with professionals only as participants[35–37] and four with both patients/families and professionals[38–41]."<br><br>The section about methods now reads: "Seventeen out of the eighteen included papers used qualitative or mixed methods. Fifteen papers used qualitative methods only (interviews and/or focus groups[24–32,35,37–41] with one study combining these with observations[35]);, one used audit[33], and two used mixed qualitative and quantitative methods (combining interviews/focus groups with surveys[34] and stakeholder workshops[36]); and one used audit[33]. ." We have also slightly reworded this question to make it clearer: "Which methods have been used to investigate communication during these contacts?"
The results are clear and resonate with my professional experience. The discussion and conclusion provide a coherent summary of the results and implications of the findings.	Thank you. It's reassuring that our analysis fits with your own experience.

Response to Reviewer 2, Dr. Maria Heckel:

Thank you for your helpful review. We have addressed your comments in the table below:

**Comments**	**Response**

There are some typos (introduction page 2 line 49-50).	I'm not certain whether I have found these as I can't seem to access the version of the manuscript with line numbers, but I have corrected any typos I have found.
The introduction does not explain why the focus is on primary care, as reflected in the inclusion criteria.	This now reads: "A key part of care in the community is out-of-hours (OOH) primary care; around one third of patients had contacts with OOH primary care services within 30 days of their death… Primary care is often the first point of contact for patients OOH and sits at the interface between specialist palliative care, secondary care, and emergency services. Good communication in OOH primary care is therefore vital to patients receiving appropriate care."
The first paragraph emphasises pain control as a major issue in palliative care. While this may be one of the most frequent problems, other symptoms could also be mentioned.	This now reads: "Getting palliative and end-of-life care right is important to ensure patients' pain, fatigue, nausea, agitation and other palliative symptoms are treated swiftly, unnecessary hospitalisations are avoided, and timely conversations are had to prepare patients and families for the patient's death."
In the methods section you mention that "Following the JBI guidance, only concepts and findings relevant to the research questions were extracted." Please specify the concepts relevant to the research questions.	We have added: "(i.e. experiences of communication in OOH primary care at the end-of-life, the barriers and facilitators to good communication, and the approaches taken to research this topic)"
I appreciate the PPI very much. I recognize that it is quite difficult to describe the impact. However, could you provide more detail about the feedback received and how it helped with the interpretation of the findings?	The PPI section now reads as follows: "Preliminary findings were presented to a group of three people with experience of OOH care for patients approaching end-of-life as family members. Initial themes were discussed in the context of their own experiences of OOH end-of-life care. This helped to validate the analysis and provided a guide for areas to look at in more detail. For instance the theme 'Out of hours professionals' manner' was developed as a theme in its own right after PPI representatives emphasised that this was at the core of how positively or negatively they recalled their experiences of OOH communication. They also highlighted the value of including quotes from participants to make the findings clearer and more powerful."
